# Early alterations of cortical thickness and gyrification in migraine without aura: a retrospective MRI study in pediatric patients

**DOI:** 10.1186/s10194-021-01290-y

**Published:** 2021-07-22

**Authors:** Alessia Guarnera, Francesca Bottino, Antonio Napolitano, Giorgia Sforza, Marco Cappa, Laura Chioma, Luca Pasquini, Maria Camilla Rossi-Espagnet, Giulia Lucignani, Lorenzo Figà-Talamanca, Chiara Carducci, Claudia Ruscitto, Massimiliano Valeriani, Daniela Longo, Laura Papetti

**Affiliations:** 1 Neuroradiology Unit, Imaging Department, Bambino Gesù Children’s Hospital, IRCCS, Piazza S. Onofrio 4, 00165 Rome, Italy; 2 Neuroradiology Unit, NESMOS Department, Sant’Andrea Hospital, La Sapienza University, Via di Grottarossa, 1035-1039, 00189 Rome, Italy; 3 Medical Physics Department, Bambino Gesù Children’s Hospital, Rome, Italy; 4 Pediatric Headache Center, Bambino Gesù Children’s Hospital, IRCCS, Piazza Sant’Onofrio 4, 00165 Rome, Italy; 5 Unit of Endocrinology, Bambino Gesù Children’s Hospital, IRCCS, Piazza Sant’Onofrio 4, 00165 Rome, Italy; 6 Neuroradiology Service, Department of Radiology, Memorial Sloan Kettering Cancer Center, 10065 New York City, NY USA; 7 Child Neurology Unit, Systems Medicine Department, Tor Vergata University Hospital of Rome, 00133 Rome, Italy; 8 Center for Sensory-Motor Interaction, Aalborg University, 9220 Aalborg, Denmark

**Keywords:** migraine, aura, magnetic resonance imaging, cortical thickness, local gyrification index, cortical morphometry, cortical abnormalities, pediatric, phonophobia

## Abstract

**Background:**

Migraine is the most common neurological disease, with high social-economical burden. Although there is growing evidence of brain structural and functional abnormalities in patients with migraine, few studies have been conducted on children and no studies investigating cortical gyrification have been conducted on pediatric patients affected by migraine without aura.

**Methods:**

Seventy-two pediatric patients affected by migraine without aura and eighty-two controls aged between 6 and 18 were retrospectively recruited with the following inclusion criteria: MRI exam showing no morphological or signal abnormalities, no systemic comorbidities, no abnormal neurological examination. Cortical thickness (CT) and local gyrification index (LGI) were obtained through a dedicated algorithm, consisting of a combination of voxel-based and surface-based morphometric techniques. The statistical analysis was performed separately on CT and LGI between: patients and controls; subgroups of controls and subgroups of patients.

**Results:**

Patients showed a decreased LGI in the left superior parietal lobule and in the supramarginal gyrus, compared to controls. Female patients presented a decreased LGI in the right superior, middle and transverse temporal gyri, right postcentral gyrus and supramarginal gyrus compared to male patients. Compared to migraine patients younger than 12 years, the ≥ 12-year-old subjects showed a decreased CT in the superior and middle frontal gyri, pre- and post-central cortex, paracentral lobule, superior and transverse temporal gyri, supramarginal gyrus and posterior insula. Migraine patients experiencing nausea and/or vomiting during headache attacks presented an increased CT in the pars opercularis of the left inferior frontal gyrus.

**Conclusions:**

Differences in CT and LGI in patients affected by migraine without aura may suggest the presence of congenital and acquired abnormalities in migraine and that migraine might represent a vast spectrum of different entities. In particular, ≥ 12-year-old pediatric patients showed a decreased CT in areas related to the executive function and nociceptive networks compared to younger patients, while female patients compared to males showed a decreased CT of the auditory cortex compared to males. Therefore, early and tailored therapies are paramount to obtain migraine control, prevent cerebral reduction of cortical thickness and preserve executive function and nociception networks to ensure a high quality of life.

## Background

Migraine is the most common neurological disease [1–3] and ranks among the most disabling medical conditions, according to the WHO (World Health Organization) [4], with significant socio-economic burden[5].

Despite its high frequency, migraine pathophysiology and structural-functional features are far from being fully understood[6].

There is growing evidence that migraine may be a progressive disorder and cause brain structural and functional alterations [7–9]. Therefore, several Magnetic Resonance studies on these abnormalities have been conducted during the last two decades. Literature has mainly focused on adult populations [6, 9] and many studies have been conducted on small cohort samples, frequently with no distinction among the different migraine subtypes[10].

Migraine is a complex disorder, that may be related to an intrinsic predisposition reflected by anomalies in cortical gyrification and disease-related processes acting on cortical thickness. Studies on children are required to clarify the precise onset and nature of underlying cortical changes [6, 11].

The investigation of pediatric migraine has frequently been encouraged to improve our understanding of the different features of the disease in children, such as duration and associated symptoms[6, 9].

To the best of our knowledge, no studies on cortical gyrification index have been conducted on children affected by migraine without aura and no studies have investigated a possible correlation between various migraine symptoms and gyrification abnormalities in pediatric populations.

The main goals of our study were to: (1) identify different patterns of cortical thickness and gyrification in pediatric patients affected by migraine without aura compared to healthy controls; (2) investigate possible correlations between these MRI parameters and clinical and demographic characteristics in patients compared to healthy controls; (3) evaluate any correlations between these MRI parameters and clinical and demographic characteristics among subgroups of patients, to identify potential brain imaging biomarkers.

## Methods

### Participants

The study was conducted in accordance with the ethical standards of the Institutional Research Committee and in accordance with the Helsinki Declaration and subsequent amendments. Informed consent was obtained from the Patients or their tutors prior to the MRI examination.

Patients were retrospectively recruited by reviewing Bambino Gesù Hospital Imaging archive from the 1st of January 2018 to the 31st of October 2020 using “headache” and “migraine” as keywords.

Two radiologists, respectively with thirty and six years of experience, performed a double-blinded analysis of patients’ MRIs and any disagreement was resolved through a consensus. We included a total of 201 patients aged between 6 and 18 years with a high quality MRI including 3D T1 MPRAGE (Magnetization Prepared Rapid Gradient Echo Imaging) sequence and showing no morphological or signal abnormalities.

An experienced neurologist reviewed patients’ medical records. Exclusion criteria were as follows: abnormal neurological examination; migraine attack during MRI; migraine prophylactic therapy; migraine abortive therapy within 24 h before the exam; catamenial migraines; sleep disorders; special abilities (athletic or artistic); major systemic disorders (psychological, oncological, vascular or other); maternal pathologies during pregnancy.

Eighty patients suffering from migraine without aura were interviewed and visited by two neurologists to confirm the diagnosis of migraine without aura according to the International Classification of Headache (ICHD3) [12] and to collect additional data.

In an anonymized database the following patients’ data were reported: demographic characteristics, laterality of migraine; migraine monthly days; personal and familiar history of migraine; pain intensity; associated symptoms (photophobia, phonophobia, nausea and vomiting).

The final cohort of patients consisted of 72 patients aged between 6 and 18 years (F:42) affected by episodic migraine without aura with a high-quality MRI exam acquired during the inter-ictal phase. The quality check of MRI examinations excluded the presence of signal and/or morphological abnormalities which may affect cortical gyrification index and thickness due to confounding factors such as WM lesions, which have been suggested to contribute to GM changes [13–15].

Eighty-two healthy subjects aged between 6 and 18 years (F:40) formed the control group with optimal age and sex matching to minimize variability [16, 17]. These subjects presented a high quality MRI exam without morphological and signal abnormalities and were interviewed and visited by a neurologist before inclusion in the control group in order to exclude migraine and other neurological diseases. No familiarity for migraine and/or headache was reported among healthy controls.

### MRI protocol

Patients and controls brain MRIs were performed on the same 3T scanner (Magnetom Skyra, Siemens, Erlangen, Germany) with a 32-channel brain coil (L-W-H: 440 mm × 330mm × 370 mm) and the following protocol: axial turbo spin-echo T2 (TR 6380 ms, TE 109ms, ST 3mm); coronal turbo spin-echo T2 (TR 6380 ms, TE 109ms, FA 150°, ST 3mm); axial FLAIR (TR 9000ms, TE 81ms, TI 2500ms, FA 150°, ST 3mm); axial DWI (TR 6400ms, TE 98ms, FA 75°, ST 4mm); sagittal 3D T1 MPRAGE (TR 1570ms, TE 2.67ms, TI 900ms, FA 9°, ST 0.8mm).

### Data processing

Data was pre-processed with FreeSurfer 5.3 software [18], using a standard automatic pipeline (i.e. recon-all) that sequentially performed skull stripping, noise, bias, intensity correction and transformation to Talairach-Tournoux space to produce grey matter (GM) and white matter (WM) segmentation. Particularly, the FreeSurfer automatic pipeline determined and tessellated the GM–WM boundary to generate the inner cortical surface (white surface), by combining information from tissue intensity and neighborhood constraints. The outer surface (pial surface) was generated through the expansion of the white surface with a point-to-point correspondence. Moreover, the FreeSurfer automatic pipeline computed Cortical thickness (CT) for each subject as the average distance measured from each surface to the other, according to Fischl and Dale [19]. The reconstructed white and grey surfaces obtained were visually checked to verify and correct any algorithmic misinterpretation of gyri and sulci. Local gyrification indices (LGI) were computed vertex-wise over the entire cortex by using the novel approach proposed by Lyu et al. (https://github.com/ilwoolyu/LocalGyrificationIndex) [20], which is based on adaptive kernel for quantification of the local cortical folding. This algorithm quantifies cortical gyrification based on a spatially-adaptive kernel, which incorporates neighboring gyral crowns and sulcal fundi.

For statistical purposes and visualization, each subject was registered on Fsaverage brain surfaces, widely used in children’s and teenagers’ studies [21–23]. Fsaverage surfaces are composed by 163,842 total vertices which represent an index of spatial resolution for analyses performed across the brain surface grid based.

### Statistical analysis

Sample sizes were estimated a priori as indicated by Pardoe et al. [24]. In particular, 50 subjects for each sample are required to detect a 0.25mm cortical thickness difference and 10 subjects per group are required to detect a 1mm cortical thickness difference [24]. Power calculation was set at 0.8 and type I error was set at p < 0.05. Univariate analysis of variance was carried out with SPSS software (PAWS Statistics 18.0) to test between-group differences in demographic variables. The statistical analysis was performed separately on CT and LGI between: healthy subjects and patients; subgroups of healthy controls and subgroups of patients (Table 1). We tested group differences in cortical parameters distributions by CT and LGI vertex-wise value mapping on a common spherical coordinate system (i.e. *fsaverage*), using spherical transformation. Differences among groups were assessed using the permutation test (1000 permutations per test) based on t statistics, performed with the Permutation Analysis of Linear Models (PALM) FSL package. Subjects age was used as covariate of no interest. Age was mean centered (across all subjects) by subtracting the overall mean age from each individual age. We also computed Threshold-Free Cluster Enhancement statistical maps, where the initial raw statistical images were enhanced using both the intensity of the data point and information from neighboring voxels[25]. Differences between groups were detected by thresholding at family-wise error (FWE), corrected at *p* < 0.05.

**Table 1:** Statistical analysis performed on Cortical Thickness and Local Gyrification Index

PATIENTS VS CONTROLS			
**Cohort 1**	** N°**	**Cohort 2**	** N°**
Patients	72	Controls	82
Patients < 12 y	42	Controls < 12 y	46
Patients ≥ 12y	30	Controls ≥ 12y	36
**SUBGROUPS OF CONTROLS**			
**Cohort 1**	** N°**	**Cohort 2**	** N°**
< 12y	46	≥ 12y	36
Females	41	Males	41
**SUBGROUPS OF PATIENTS**			
**Cohort 1**	** N°**	**Cohort 2**	** N°**
Patients < 12y	42	≥ 12y	30
Females	42	Males	30
Patients with MMD < 5	42	Patients with MMD ≥ 5	30
Patients with photophobia and phonophobia	41	Patients with photophobia or phonophobia	62
Patients with photophobia	50	Patients without photophobia	22
Patients with nausea and/or vomiting	38	Patients without nausea and/or vomiting	34

*The table illustrates the statistical analysis performed separately on cortical thickness and gyrification between: healthy patients and patients; subgroups of healthy controls and subgroups of patients. Cohort 1 and cohort 2 indicate the two groups of patients compared for each statistical analysis and n° indicates the number of patients forming a particular cohort.*

*MMD: migraine monthly days.*

## Results

The main demographic and clinical characteristics of patients with migraine and healthy control subjects are summarized in Table 2. Neither sex distribution (*P* = 0.2) nor mean age (*P* = 0.2) significantly differed between patients with migraine and healthy control subjects. Results were corrected in order to avoid false positives [10, 16, 17].

**Table 2:** Main demographic and clinical characteristics of healthy controls and patients affected by migraine without aura. Neither gender distribution (*P* = 0.2) nor mean age (*P* = 0.2) significantly differed between patients with migraine and healthy control subjects

Characteristics	Healthy Controls	Patients
N° of Subjects	82	72
N° of Subjects < 12 years	46	42
N° of Subjects ≥ 12 years	36	30
N° F/M	41/41	42/30
Mean Age in years (STD)	10.96 (3.75)	11.73 (3.19)
N° of Subjects presenting MMG < 5	-	42
N° of Subjects presenting MMG ≥ 5	-	30
Migraine Duration per attack in hours (N° of patients)	-	< 2 h (26), <4 h (27), < 72 h (19)
Months since first attack (n° of patients)	-	< 6 (3), < 12 (12), < 24 (13), < 36 (19), < 72 (25)
Pain Intensity	-	mild (20), moderate (22), severe (30)
N° of Subjects presenting Photophobia	-	50
N° of Subjects presenting Phonophobia	-	53
N° of Subjects presenting Nausea	-	35
N° of Subjects presenting Vomiting	-	16
N° of Subjects presenting familiar cases of migraine	-	None (9), Mother (35), Father (7), Both parents (21)

*F/M: females/males; STD: standard deviation; MMD: migraine monthly days.*

### Cortical Gyrification

**Fig. 1:**
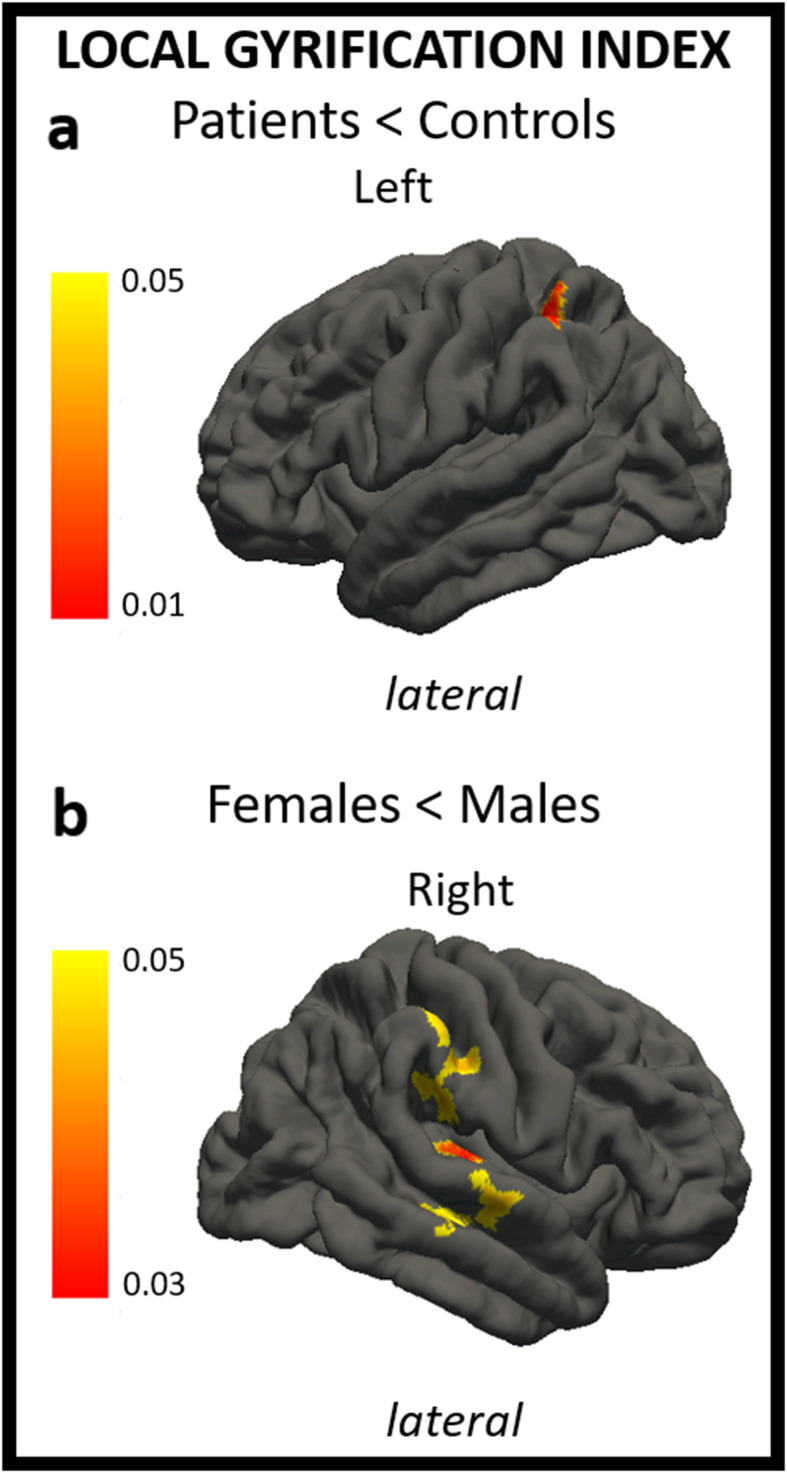
The figure shows differences in local gyrification index in patients compared to healthy controls (**A**) and in female patients compared to male patients (**B**). In a, regions of decreased local gyrification index in patients vs. controls are shown through a color scale ranging from yellow (*p* < 0.05) to red (*p* < 0.01). In b, regions of decreased local gyrification index in females vs. males are shown through a color scale ranging from yellow (*p* < 0.05) to red (*p* < 0.03). Only the most representative views are shown.

Patients showed a decreased gyrification index in the left superior parietal lobule and in the left inferior parietal lobule, particularly in the supramarginal gyrus, compared to healthy controls (p < 0.05). We found a decreased gyrification index in the right superior, middle and transverse temporal gyri, in the right postcentral gyrus and in the right supramarginal gyrus in females compared to male patients (p < 0.05) (Table 3). Statistical results including p-value maps are displayed on a common surface template in Fig. 1.

**Table 3:** Results obtained from the statistical analysis performed on Cortical Thickness and Local Gyrification Index

LOCAL GYRIFICATION INDEX					
**Patients vs. Controls**	**Patients < Controls**	**n° Vertices**	**Mean p value**	**Controls Mean LGI (STD)**	**Patients Mean LGI (STD)**
	*Left supramarginal gyrus*	1262	0.01	3.53 (2.40)	3.51 (2.33)
	*Left superior parietal gyrus*	1014	0.01	3.68 (2.57)	3.65 (2.52)
	*Left inferior parietal gyrus*	256	0.02	4.03 (2.74)	4.00 (2.66)
**Subgroups of Patients**	**Females < Males**	**n° Vertices**	**Mean p value**	**F Mean LGI (STD)**	**M Mean LGI (STD)**
	*Right postcentral gyrus*	187	0.05	3.40 (1.81)	3.49 (1.88)
	*Right supramarginal gyrus*	888	0.05	3.25 (1.79)	3.30 (1.87)
	*Right superior temporal gyrus*	724	0.04	3.41 (2.09)	3.47 (2.17)
	*Right middle temporal gyrus*	111	0.05	3.95 (2.67)	4.04 (2.75)
	*Right transverse temporal gyrus*	405	0.04	3.30 (2.01)	3.37 (2.09)
**CORTICAL THICKNESS**					
**Subgroups of Patients**	**Patients < 12y > Patients ≥ 12y**	**n° Vertices**	**Mean p value**	**Patients < 12y Mean CT (STD) in mm**	**Patients ≥ 12y Mean CT (STD)**
	*Left superior frontal gyrus*	356	0.02	2.43 (0.98)	2.36 (0.96)
	*Left middle frontal gyrus*	434	0.02	2.78 (0.67)	2.71 (0.65)
	*Left precentral gyrus*	3914	0.02	2.74 (0.73)	2.67 (0.71)
	*Left postcentral gyrus*	705	0.02	2.67 (0.74)	2.61 (0.72)
	*Left paracentral lobule*	1186	0.02	2.61 (0.85)	2.54 (0.83)
	*Left superior temporal gyrus*	739	0.03	2.73 (0.74)	2.65 (0.72)
	*Left transverse temporal gyrus*	427	0.03	2.66 (0.78)	2.58 (0.76)
	*Left supramarginal gyrus*	523	0.03	2.71 (0.80)	2.64 (0.79)
	*Left posterior insula*	361	0.03	2.64 (0.74)	2.56 (0.72)
	**Patients with nausea and/or vomiting > Patients without nausea and/or vomiting**	**n° Vertices**	**Mean p value**	**Patients without nausea and/or vomiting Mean CT (STD) in mm**	**Patients with nausea and/or vomiting Mean CT (STD)**
	*Left pars opercularis*	113	0.04	2.52 (0.94)	2.48 (0.92)

*The table illustrates the results from statistical analysis performed separately on cortical thickness and gyrification between: healthy patients and patients; subgroups of healthy controls and subgroups of patients.*

*LGI: local gyrification index; CT: cortical thickness; mm: millimetres.*

### Cortical Thickness

**Fig. 2:**
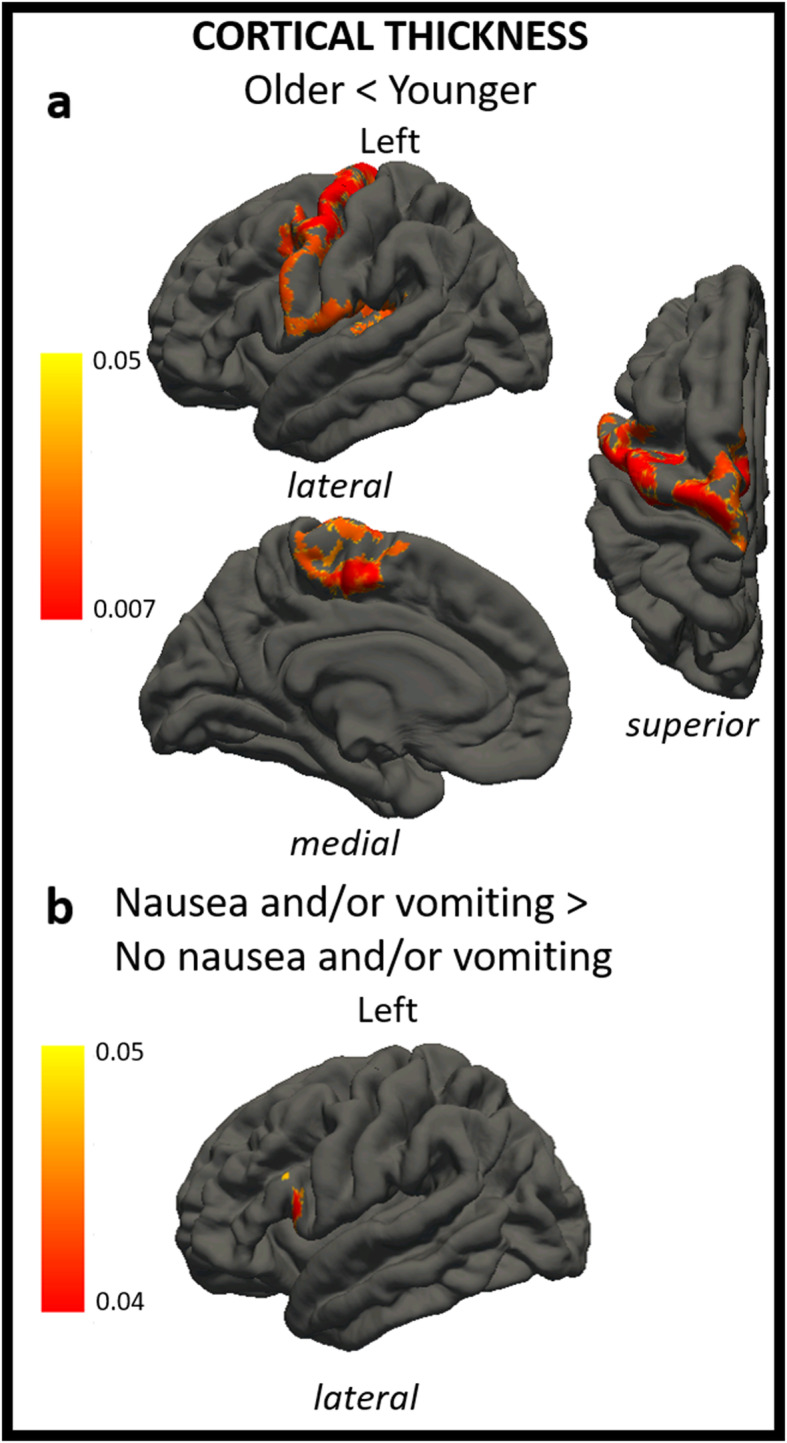
The figure shows differences in cortical thickness in ≥ 12-year-old patients compared to < 12-year-old patients (**A**) and in patients with nausea and/or vomiting compared to patients without nausea and/or vomiting (**B**). In a, regions of decreased cortical thickness in ≥ 12-year-old patients vs. < 12-year-old patients are shown through a colour scale ranging from yellow (*p* < 0.05) to red (*p* < 0.007). In b, regions of increased cortical thickness in patients with nausea and/or vomiting vs. patients without nausea and/or vomiting are shown through a colour scale ranging from yellow (*p* < 0.05) to red (*p* < 0.007). Only the most representative views are shown.

Patients ≥ 12-year-old showed decreased cortical thickness compared to younger ones, particularly involving: superior and middle frontal gyri, pre- and post-central cortex, paracentral lobule, superior and transverse temporal gyri, supramarginal gyrus and posterior insula. Migraine patients experiencing nausea and/or vomiting during headache attacks presented an increased cortical thickness in the pars opercularis of the inferior frontal gyrus (Table 3). Statistical results including p-value maps are displayed on a common surface template in Fig. 2.

## Discussion

We found abnormalities in local gyrification index and cortical thickness in patients affected by migraine without aura compared to healthy controls and among patient subgroups. This evidence supports the idea of migraine being a complex pathology, possibly related to congenital and acquired brain abnormalities presenting from a very young age.

The working theory describes migraine pathogenesis as related to hyperexcitability of the trigeminovascular system. Specifically, cortical spreading depressions may promote the release of molecular mediators in the dura resulting in meningeal inflammation and sensory excitation of trigeminal afferences. This nociceptive information is then projected on multiple cortical areas through central brain regions, leading to migraine symptoms [26].

Although its specific role in these processes is far from being fully understood, the cortex has been linked to the modulation and representation of pain in migraine as well as to the amplification of sensory inputs [27].

Neuroimaging studies have demonstrated that migraine patients present functional and morphological abnormalities compared to controls in relation to variables such as attack frequency or migraine attack duration, suggesting a possible link between anatomical and brain changes [28–30]. In particular, neuroimaging studies demonstrated macroscopic and microscopic changes in the brain of migraine patients, together with functional networks modifications [31].

### Local Gyrification Index

Cortical gyrification is a complex process, which mainly takes place during the late fetal development and consists in cerebral cortex folding to allow cortical surface increase [11, 32, 33]. As it remains essentially stable during life, differences in cortical gyrification may reflect a congenital predisposition to develop migraine.

Despite the crucial information that cortical gyrification may offer, only two studies have investigated gyrification index changes in small cohorts of adults affected by migraine without aura[5, 10]. In particular, Zhang et al. [5] identified an increased gyrification index in left postcentral gyrus, superior parietal lobule and right lateral occipital cortex, and a decreased gyrification index in the left rostral middle frontal gyrus compared with controls, while no differences have been identified by Masson et al. [10]. Moreover, Rieder et al. [34] investigated cortical alterations in medication-overuse headache in adults and observed higher LGI in two clusters extending respectively from the fusiform gyrus to adjacent medial temporal regions and in the occipital pole, this last being a good predictor for poor response after detoxification. On the other hand, Lai et al. [35] did not find significant differences between patients affected by chronic migraine and controls. These results, particularly from the study of Rieder et al. [34], may suggest a neurodevelopmental component to migraine disease chronicization or genetic predisposition to a more severe disease type.

#### Patients vs. healthy controls

We found a decreased gyrification index in the left superior parietal lobule and in the left inferior parietal lobule, particularly in the supramarginal gyrus, of patients compared to healthy controls.

The superior and the inferior parietal lobule are included in the [36]executive control network, which mainly involves fronto-striatal-parietal brain regions. Executive functions represent a complex subgroup of cognitive functions, which ensure a finalized behavior in multiple-choice contexts [36–38]. In particular, the superior parietal lobule has a pivotal role in task-switching, set-shifting and in the integration of information [39], while the inferior parietal lobule monitors selective attention [36]. The finding of a decreased gyrification index in the superior and inferior parietal lobule could explain the executive function deficits found in migraine patients during both ictal and interictal phases [37].

The superior and the inferior parietal lobule play a key role in the nociceptive pathway, particularly, the primary and secondary somatosensory cortices form the lateral pain system. Also, they add sensory-discrimination properties to pain processing by encoding location, intensity, and quality of pain [40–42]. This information is projected to the posterior parietal cortex which provides modulatory influences and conveys nociceptive information to widespread cortical brain regions [3, 42, 43]. The supramarginal gyrus is specifically involved in the cognitive evaluation of pain [44, 45] and a reduced pain-related activity of the supramarginal gyrus has been reported in headache patients with medication overuse [46].

The presented results confirm the longitudinal study of Liu.et al. [3], which assessed GM changes at baseline and after a follow-up of 1 year, showing decreased GM in the superior parietal gyrus, inferior parietal gyrus and supramarginal gyrus of migraine patients at follow-up. Structural changes in inferior parietal lobule and supramarginal gyrus match functional abnormalities identified in chronic migraine patients in a fMRI study by Chiapparini et al. [42] and functional alterations and abnormal connectivity identified between these areas and the hypothalamus in an fMRI study on cluster-headache by Ferraro et al. [46]. In particular, Chiapparini et al. tested brain areas activation during mechanical painful stimuli administration in chronic migraine patients and demonstrated significant activations in the inferior parietal cortex and in the supramarginal gyrus and reduced pain-related activity in the lateral pain pathway of patients with headache medication overuse [42].

These findings may suggest a role for these brain regions in migraine pain processing. In particular, an abnormal gyrification index in the superior and inferior parietal lobules may alter patients’ pain sensory-discrimination abilities and the complex modulatory mechanism taking part in pain processing, possibly disrupting the cognitive evaluation of pain.

#### Female migraine vs. male migraine

We found significantly decreased gyrification index in the right superior, middle and transverse temporal gyri, in the right postcentral gyrus and in the right supramarginal gyrus of females compared to male patients.

The search for a “sex specific phenotype” [47] of migraine is justified by the increased prevalence of migraine in females over 12 years [48, 49], differences in clinical symptoms and migraine presentation between the sexes [50, 51], the potential role of female hormones in migraine onset [52] and response to treatment [53].

Recent literature suggested that female and male migraine may be different in relation to specific functional and structural brain abnormalities compared to controls [6, 47, 54–56].

As already discussed above, postcentral and supramarginal gyri are included in the nociceptive pathway. Previous studies investigating gray matter cortical thickness showed that females present an increased cortical thickness in areas associated with nociception, including the somatosensory cortex [54]. In particular, somatosensory cortical thickness has been demonstrated to be negatively correlated with response to migraine medications [55].

Although the role of the temporal lobes in pain processing is not completely understood and few studies are available in literature regarding temporal lobe abnormalities in migraine [57, 58], these areas are supposed to assign emotional valence to short-term memories related to painful experiences [59]. In particular, the superior temporal gyrus is involved in pain processing by supervising the mismatch between pain expectation and pain perception [60], pain anticipation [61], and pain expression [62]. In a fMRI study, Schwedt et al. [1] showed atypical connectivity between the middle temporal gyrus and widespread subcortical and cortical areas in migraine patients, possibly reflecting abnormal pain processing.

Our results confirm the hypothesis presented by Webb et al. [6], who suggested that structural abnormalities indicate the importance of sex in migraine.

Superior temporal gyrus and Heschl’s gyrus, in particular Broadman areas 41–42 and partially 22, form the primary auditory cortex [63, 64]. An increased volume of these gyri, as described by Aldemir et al. [57], may partially explain patient deficits in auditory stimuli processing, characterized by hypersensitivity and aversion to sound during migraine attacks, the onset of migraine attacks with auditory triggers, and the atypical sensory perception during the interictal phase [65, 66].

Differences in the gyrification index of the auditory cortex may justify different clinical presentations between male and female patients and why male patients with migraine more frequently present phonophobia compared to female ones [48, 50].

### Cortical Thickness

CT undergoes dynamic changes through life in relation to normal development and diseases [11, 32, 33]. Since CT displays some variability due to physiologic and pathologic processes, changes induced by migraine appear realistic. Although cortical thickness in adult [14, 36, 47] and pediatric [11, 58] migraine patients has been widely investigated, no “reliable brain morphological signature for migraine” [67] has been demonstrated so far.

#### < 12-year-old Patients vs. ≥ 12-year-old Patients with migraine

We found cortical thickness to be significantly reduced in migraine patients ≥ 12 years compared to those < 12 years in: superior and middle frontal gyri, pre- and post-central cortex, paracentral lobule, superior and transverse temporal gyri, supramarginal gyrus and posterior insula.

Pediatric patients were divided in two subgroups in relation to age, since migraine prevalence of both sexes is almost equal up to age 12 and there are no sex-related differences, while after age 12 there is a significant female predominance, which progressively increases with age [68, 69].

A decreased cortical thickness in patients ≥ 12 years may be justified by the older age and the longer duration of the disease compared to < 12-years-old patients. This evidence is coherent with the inverse correlation [70, 71]between cortical thickness and age in migraine patients .

The finding of reduced cortical thickness of both frontal lobes in migraine patients is supported by several studies conducted on children [11, 13, 58] and adults [14, 36, 72, 73]. As discussed above, this finding can be related to deficits in the executive control network during both ictal and interictal phases [37].

A decreased cortical thickness in the precentral, postcentral and supramarginal gyri confirms prior results obtained in studies conducted on adult migraine patients [3, 14, 71, 74]. In particular, these cortical areas cooperate in pain processing by modulating the cognitive dimension of pain and by encoding the expected painful stimulus in relation to spatial location, intensity and quality of pain [14, 40–42, 75, 76]. The result obtained in the postcentral gyrus is supported by previous studies that show loss of volume and thinning of the cortex in patients with chronic pain of non-migraine origin [77, 78]. On the other hand, the superior temporal gyrus is involved in pain processing by supervising the mismatch between pain expectation and pain perception [60], and by regulating pain anticipation [61] and expression [62].

It has been suggested that frontal cortex reduction of cortical thickness is associated with the reorganization of the nociceptive network in relation to pain processing of migraine [14, 58, 73]. However, the temporal relation between migraine and cortical reduction of cortical thickness is not yet clear [6].

The identification of a thinner cortex in the superior and transverse temporal gyri may reflect the aforementioned deficits in auditory stimuli processing which are mostly appreciable during migraine attacks, but may or could persist in the interictal phase [65, 66].

The finding of a reduced cortical thickness in the posterior insula confirms prior literature. The pivotal role of insula in migraine and in the nociceptive network led to defining[80] insula as a “hub of activity” in migraine [79].

The insula represents a “multidimensional integration site for pain” since the nociceptive input received from the trigeminovascular pathway via the thalamus is first processed in the posterior insula, which encodes the intensity of pain [71, 81–84] and its anatomical location. From the posterior insula, the nociceptive input is conveyed to the anterior insula, which assigns emotional significance to the painful stimulus [79]. fMRI and PET studies have demonstrated posterior insula contribution to the sensory-discriminative aspects of pain processing [85–89], as it presents strong connectivity with the premotor, sensorimotor and supplementary motor areas and with the middle-posterior cingulate cortex [79, 90].

The posterior insula is also involved in interoceptive awareness, related to the internal state of migraine patients, which changes between the ictal and interictal phase [79, 91].

The finding of thinner insular cortex in patients ≥ 12 years is supported by connectivity studies, as well as the evidence of structural-functional alterations in the pain processing network with aging. In particular, Dennis et al. [92] showed modifications in fiber density between the insula and various cortical regions from 12 to 30 years of age, while Ceko et al. [93] demonstrated a functional shift in insular connectivity in chronic pain states from being adaptive in < 12-year-old patients to being maladaptive in ≥ 12-year-old ones. Insular changes with aging result in structural and functional alterations in pain processing [79, 94].

Accordingly, decreased cortical thickness of the posterior insula may reflect a structural-functional imbalance of brain homeostasis and may cause an altered perception and processing of pain [72, 95].

Insular involvement in migraine pathophysiology and the evidence of insular structural and functional alterations may pave the way to new and non-invasive treatment approaches in pediatric migraine. Particularly, aerobic exercise [96], stress limitation, maintenance of good hydration [97], optimal sleep hygiene [79], cognitive behavioral therapy along with pharmacotherapy have been proved extremely successful in migraine patients [79].

The importance of a tailored therapy roots in crucial differences between pediatric and adult migraine, as demonstrated by failure of standard adult treatment in the pediatric population [6, 98].

#### Patients with nausea and/or vomiting vs. patients without nausea and/or vomiting

Interestingly, patients experiencing nausea and/or vomiting during headache attacks showed increased cortical thickness in the left pars opercularis of the inferior frontal gyrus. The frontal operculum together with the insula represents the gustatory cortex, which is the cortical area dedicated to perceiving and distinguishing tastes [99]. Few studies identified abnormalities in cortical thickness of pars opercularis in patients affected by migraine and no paper has correlated changes in this area with migraine symptoms such as nausea or vomiting. In particular, Planchuelo-Gomez et al. [100] identified a significant negative correlation between attack duration in episodic migraine patients and gray matter volume in the right pars opercularis, while Hougaard et al. made a between-hemisphere comparison in migraine patients and identified an increased cortical thickness in the pars opercularis contralateral to the perceived headache side.

Harriott et al. [65] suggested that migraine patients experiment altered perception of painful and non-painful stimuli during ictal and interictal phases (unimodal special sensory processing in migraine patients) and present an abnormal integration of information from simultaneous and different sensory inputs (multisensory processing and integration).

Increased cortical thickness in the pars opercularis may reflect altered perception of stimuli and altered integration of information, leading to the experience of nausea and vomiting during migraine attacks.

Future longitudinal studies on larger cohorts are needed to confirm the presented results.

## Limitations

The main limitation of our study is the retrospective design, which required us to select the study population from our Institution archive, including patients whose medical evaluation required a neuroimaging examination. To mitigate this limitation, we applied strict inclusion and exclusion criteria for patients and controls and interviewed and visited every subject before inclusion in the current study. Patients were not checked for anxiety or mood disorder symptoms with recognized standard scales. To limit this caveat, patients and families were interviewed to exclude physical and psychiatric comorbidities and sleep disorders.

## Conclusions

The evidence of differences in cortical thickness and local gyrification index in patients compared to controls suggests that migraine is a complex pathology, possibly related to congenital and acquired brain abnormalities. In particular, ≥ 12-year-old pediatric patients showed decreased cortical thickness in areas related to executive functions and nociceptive networks compared to < 12-year-old patients, similar to what described in adult patients. Female patients compared to males showed decreased cortical thickness of the auditory cortex, which may justify the increased prevalence of phonophobia in males. Therefore, therapies should differ among patients depending on migraine features and individual characteristics.

## Data Availability

Data and materials used and analyzed during the current study are available from the corresponding author upon reasonable request.

## Abbreviations

MRI: magnetic resonance imaging
MPRAGE: Magnetization Prepared Rapid Gradient Echo Imaging
FLAIR: Fluid Attenuated Inversion Recovery
DWI: Diffusion Weighted Image
TR: repetition time
TE: echo time
TI: inversion time
FA: flip angle
ST: slice thickness
CT: cortical thickness
LGI: local gyrification index
GI: gyrification index
WM: white matter
GM: grey matter
MMD: migraine monthly days
ICHD3: International Classification of Headache
WHO: World Health Organization
PALM: Permutation Analysis of Linear Models
FEW: Family Wise Error
STD: Standard Deviation
